# Dataset of Phenology of Mediterranean high-mountain meadows flora (Sierra Nevada, Spain)

**DOI:** 10.3897/phytokeys.46.9116

**Published:** 2015-02-27

**Authors:** Antonio Jesús Pérez-Luque, Cristina Patricia Sánchez-Rojas, Regino Zamora, Ramón Pérez-Pérez, Francisco Javier Bonet

**Affiliations:** 1 Laboratorio de Ecología (iEcolab), Instituto Interuniversitario de Investigación del Sistema Tierra en Andalucía (CEAMA), Universidad de Granada, Avenida del Mediterráneo s/n, 18006, Granada, Spain; 2 Grupo de Ecología Terrestre, Departamento de Ecología, Universidad de Granada, Facultad de Ciencias, Campus de Fuentenueva s/n, 18071, Granada, Spain; 3 Agencia de Medio Ambiente y Agua de Andalucía. Consejería de Medio Ambiente y Ordenación del Territorio. Junta de Andalucía, C/ Joaquina Egüaras, 10, 18003, Granada, Spain

**Keywords:** Wet high-mountain meadows, abundance, phenology, Sierra Nevada (Spain), long-term research, global change monitoring, occurrence, observation

## Abstract

Sierra Nevada mountain range (southern Spain) hosts a high number of endemic plant species, being one of the most important biodiversity hotspots in the Mediterranean basin. The high-mountain meadow ecosystems (*borreguiles*) harbour a large number of endemic and threatened plant species. In this data paper, we describe a dataset of the flora inhabiting this threatened ecosystem in this Mediterranean mountain. The dataset includes occurrence data for flora collected in those ecosystems in two periods: 1988–1990 and 2009–2013. A total of 11002 records of occurrences belonging to 19 orders, 28 families 52 genera were collected. 73 taxa were recorded with 29 threatened taxa. We also included data of cover-abundance and phenology attributes for the records. The dataset is included in the Sierra Nevada Global-Change Observatory (OBSNEV), a long-term research project designed to compile socio-ecological information on the major ecosystem types in order to identify the impacts of global change in this area.

## Project details

### Project title

Sierra Nevada Global-Change Observatory (OBSNEV)

### Personnel

Regino Jesús Zamora Rodríguez (Scientific Coordinator, Principal Investigator, University of Granada); Francisco Javier Sánchez Gutiérrez (Director of the Sierra Nevada National Park and Natural Park).

### Funding

Sierra Nevada Global Change Observatory is funded by Andalusian Regional Government (via Environmental Protection Agency) and by the Spanish Government (via “Fundación Biodiversidad”, which is a Public Foundation).

### Study area descriptions/descriptor

**Figure 1.**
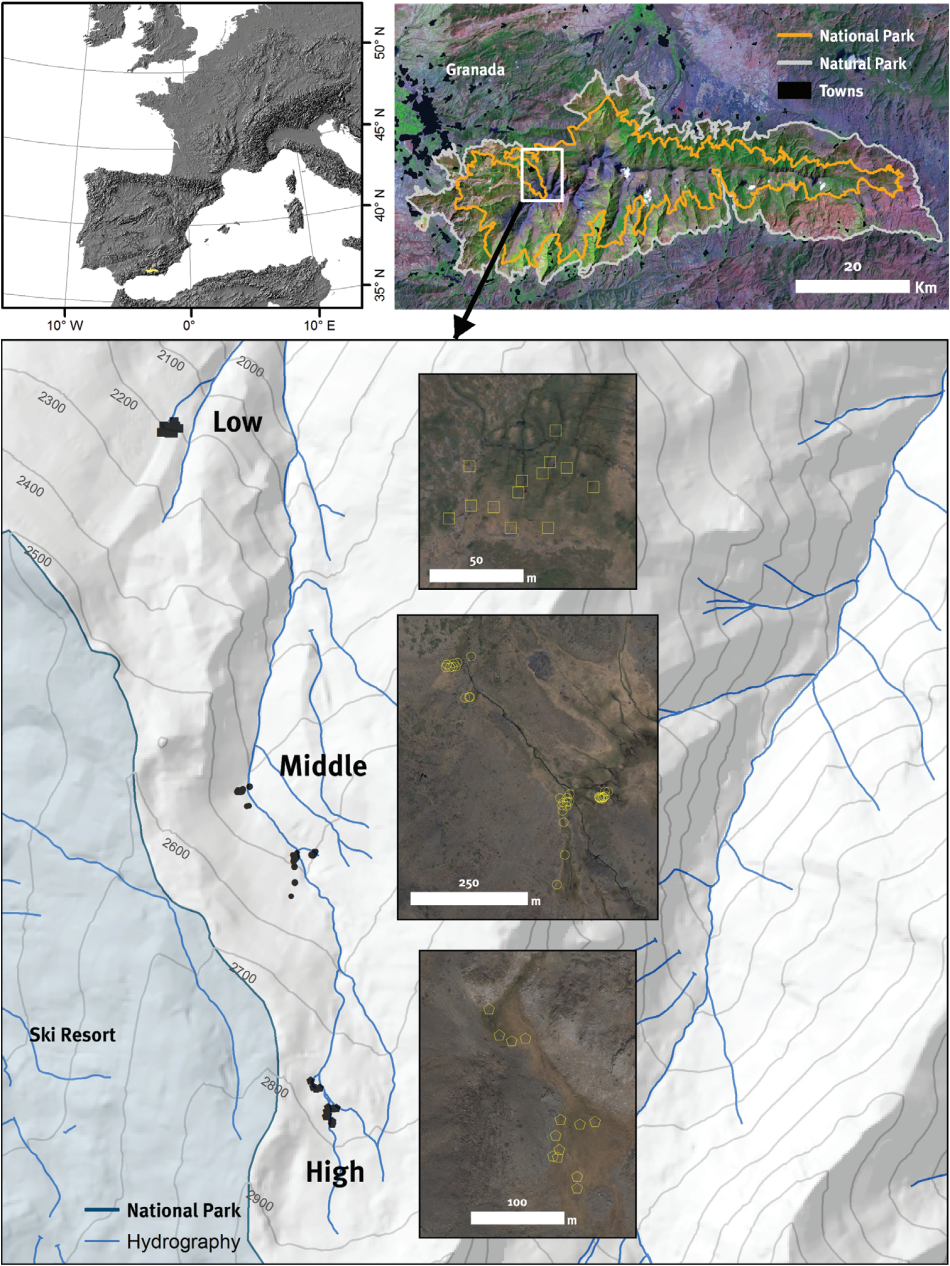
Location of Sierra Nevada (southern Spain) and boundaries of the National and Natural Parks (top panels). The bottom panel shows the location of the borreguiles in the San Juan river basin with the sampling plots along an altitudinal gradient.

Sierra Nevada (Andalusia, SE Spain), a mountainous region with an altitudinal range between 860 m and 3482 m a.s.l., covers more than 2000 km^2^ (Figure 1a, b). The climate is Mediterranean, characterized by cold winters and hot summers, with pronounced summer droughts (July–August). The annual average temperature decreases in altitude from 12–16 °C below 1500 m to 0 °C above 3000 m a.s.l., and the annual average precipitation is approximately 600 mm. Additionally, the complex orography of the mountains causes strong climatic contrasts between the sunny, dry south-facing slopes and the shaded, wetter north-facing slopes. Annual precipitation ranges from less than 250 mm in the lowest parts of the mountain range to more than 700 mm in the summit areas. Winter precipitation is mainly in the form of snow above 2000 m of altitude. The Sierra Nevada mountain range hosts a high number of endemic plant species (ca. 80; Lorite et al. 2007) for a total of 2100 species of vascular plants (25% and 20% of Spanish and European flora, respectively). This mountain area comprises 27 habitat types from the habitat directive. It contains 31 animal species (20 birds, 5 mammals, 4 invertebrates, 2 amphibians and reptiles) and 20 plant species listed in the Annex I and II of habitat and bird directives. It is thus considered one of the most important biodiversity hotspots in the Mediterranean region (Blanca 1996, Blanca et al. 1998, Cañadas et al. 2014).

This mountain range has several types of legal protection: Biosphere Reserve MAB Committee UNESCO; Special Protection Area and Site of Community Importance (Natura 2000 network); and National Park. The area includes 61 municipalities with more than 90000 inhabitants. The main economic activities are agriculture, tourism, cattle raising, beekeeping, mining, and skiing (Bonet et al. 2010).

### Design description

Sierra Nevada Global Change Observatory (OBSNEV) (Bonet et al. 2011) is a long-term research project which is being undertaken at Sierra Nevada Biosphere Reserve (SE Spain). It is intended to compile the information necessary for identifying as early as possible the impacts of global change, in order to design management mechanisms to minimize these impacts and adapt the system to new scenarios (Aspizua et al. 2010, Bonet et al. 2010). The general objectives are to:

- Evaluate the functioning of ecosystems in the Sierra Nevada Nature Reserve, their natural processes and dynamics on a medium-term time scale.
- Identify population dynamics, phenological changes, and conservation issues regarding key species that could be considered indicators of ecological processes.
- Identify the impact of global change on monitored species, ecosystems, and natural resources, providing an overview of trends of change that could help bolster ecosystem resilience.
- Design mechanisms to assess the effectiveness and efficiency of management activities performed in the Sierra Nevada in order to implement an adaptive management framework.
- Help to disseminate information of general interest concerning the values and importance of Sierra Nevada.

The Sierra Nevada Global-Change Observatory has four cornerstones:

1. A monitoring program with 40 methodologies that collect information on ecosystem functioning (Aspizua et al. 2012, 2014).
2. An information system to store and manage all the information gathered (http://obsnev.es/linaria.html – Pérez-Pérez et al. 2012; Free access upon registration).
3. A plan to promote adaptive management of natural resources using the data amassed through the monitoring programme.
4. An outreach programme to disseminate all the available information to potential users (see News Portal of the project at http://obsnev.es and the wiki of the project at http://wiki.obsnev.es, Pérez-Luque et al. 2012)

The Sierra Nevada Global Change Observatory is linked to other national (Zamora and Bonet 2011) and international monitoring networks: GLOCHAMORE (Global Change in Mountain Regions) (Björnsen 2005), GLOCHAMOST (Global Change in Mountain Sites) (Schaaf 2009), LTER-Spain (Long-Term Ecological Research). This Observatory is also involved in several European projects like MS-MONINA (FP7 project, www.ms-monina.eu) or EU BON (Hoffmann et al. 2014)

In addition to monitoring the ecosystems of this mountain range (i.e. collection of recent data from biotic and abiotic variables) the Sierra Nevada Global-Change Observatory is incorporating historical information of biodiversity into its information system and some historical experiments and studies are being revisited to detect potential changes due to global change. The dataset described here is a good example of this idea: a singular ecosystem was revisited and resampled 30 years after its inception to check whether the phenology of its flora community had undergone changes.

### Data published through GBIF

http://www.gbif.es:8080/ipt/resource.do?r=borreguiles

## Taxonomic coverage

**Figure 2.**
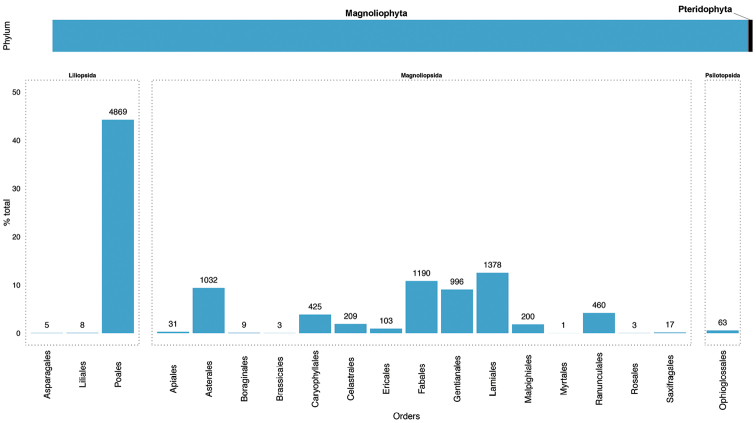
Taxonomic coverage. The upper bar shows the percentage of records of the dataset belonging to each phylum. The bottom bars show the percentage of total records in the dataset by order. The number of records is included above the bars. The order bars is aggregated by class.

**Figure 3.**
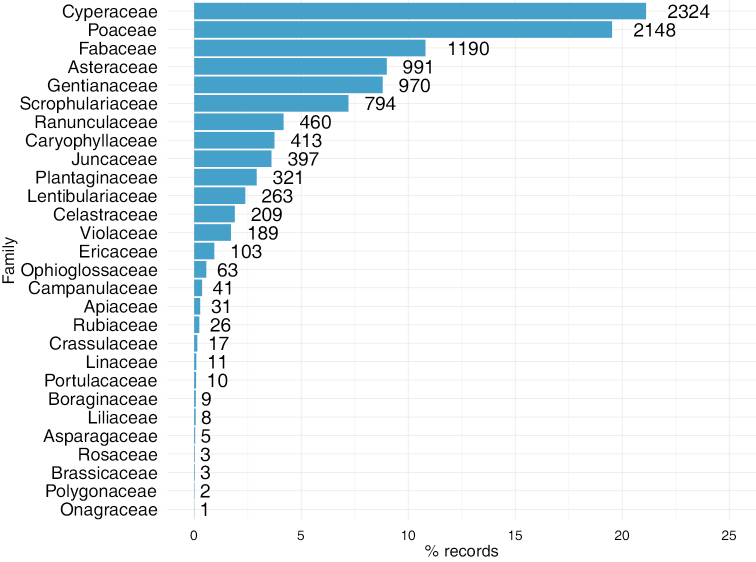
Taxonomic coverage (families). Percentage of dataset records by families. The numbers indicate the records of each family.

This dataset includes records of the phylum Magnoliophyta (10939 records, 99.43%) and marginally Pteridophyta (63 records, below 1% of total records). Most of the records included in this dataset belong to both the class Magnoliopsida (6057 records; 55.04%) and Liliopsida (4883 records; 44.37%). The class Psilotopsida is represented by 63 records. There are 19 orders represented in the dataset, Poales (44.25%) and Lamiales (12.52%) being the most important order from classes Liliopsida and Magnoliopsida, respectively (Figure 2). The class Psilotopsida is represented only by order Ophioglossales. In this collection, 28 families are represented, with Cyperaceae, Poaceae and Fabaceae being the families with highest number of records (Figure 3). The dataset contains 72 taxa belonging to 51 genera. *Carex*, *Nardus*, and *Scorzoneroides* are the most represented genera in the database. There are 29 threatened taxa (Table 1).

**Table 1.** Threatened and/or endemic species of the dataset

Scientific name	Bern ^a^	Habitat Directive ^b^	Spanish Red List ^c^	Andalusian Red List ^d^	IUCN Global ^e^	IUCN SN ^f^	Endemic ^g^
Agrostis canina L. subsp. granatensis Romero García, Blanca & C. Morales			VU	VU	VU	VU	SN
*Agrostis nevadensis* Boiss.							SN
*Arenaria tetraqueta* L.							SN
*Botrychium lunaria* (L.) Sw.				VU		VU	
*Carex capillaris* L.				DD			
*Carex nevadensis* Boiss. & Reut.				NT			
Cerastium alpinum L. subsp. aquaticum (Boiss.) Mart. Parras & Molero Mesa							SN
*Draba lutescens* Coss.				VU	LR-nt	VU	
*Eleocharis quinqueflora* (Hartmann) O. Schwarz				VU			
*Eryngium glaciale* Boiss.				NT			SN
*Euphrasia willkommii* Freyn				NT			
*Festuca frigida* Hack.			VU	VU	VU	VU	SN
*Galium nevadense* Boiss. & Reut.				NT			
*Gentiana alpina* Vill.				VU	VU	VU	
*Gentiana boryi* Boiss.			VU	VU	VU	VU	
Gentiana pneumonanthe L. subsp. depressa (Boiss.) Rivas Mart., A. Asensi, Molero Mesa & F.Valle			VU	VU	VU	VU	SN
*Gentiana sierrae* Briq.			VU	VU	VU	VU	SN
*Gentianella tenella* (Rottb.) Harry Sm.				DD		VU	
*Herniaria boissieri* J.Gay				NT			SN
Linaria aeruginea (Gouan) Cav. subsp. nevadensis (Boiss.) Rivas Mart., A. Asensi, Molero Mesa & F.Valle							SN
Lotus corniculatus L. subsp. glacialis (Boiss.) Valdés				NT			
*Luzula spicata* (L.) DC. in Lam. & DC				NT		LR-lc	
*Parnassia palustris* L.				NT			
Phleum brachystachyum (Salis) Gamisans, Romero García & C.Morales subsp. abbreviatum (Boiss.) Gamisans, Romero García & C.Morales			VU	VU	VU	VU	
*Pinguicula nevadensis* (H.Lindb.) Casper	Appendix I	Annex II	EN	VU	VU	VU	SN
*Plantago nivalis* Jord.							SN
*Potentilla nevadensis* Boiss.				NT			SN
*Ranunculus acetosellifolius* Boiss.				NT			SN
Ranunculus angustifolius DC. subsp. uniflorus (Boiss.) Molero Mesa & Pérez Raya			VU	NT			SN
*Scorzoneroides microcephala* J.Holub	Appendix I	Annex II	EN	VU	VU	VU	SN
*Scorzoneroides nevadensis* (Lange) Greuter							SN
*Thlaspi nevadense* Boiss. & Reut.			VU	VU	VU	VU	SN
Vaccinium uliginosum subsp. nanum (Boiss.) Rivas Mart., A. Asensi, Molero Mesa & F. Valle							SN
*Veronica nevadensis* H.Lindb.				DD			SN
*Viola crassiuscula* Bory				NT			SN
*Viola palustris* L.				NT			

a Bern: Convention on the Conservation of European Wildlife and Natural Habitats (Bern Convention).

b Species included in the Habitat Directive Annex (EC 1992).

c 2010 Red List of Spanish vascular flora (Moreno 2010).

d 2005 Red List of vascular flora of Andalusia (Cabezudo et al. 2005).

e IUCN category in the distribution area (Blanca et al. 2001, Lorite et al. 2007).

f IUCN category in Sierra Nevada (Blanca et al. 2001).

g Endemicity (Blanca et al. 2001).

EN Endangered

VU Vulnerable

NT Near threatened

**LR-nt:** Lower Risk-Near Threatened

**LR-cd:** Lower Risk-Conservation Dependet

**LR-lc:** Lower Risk-Least Concern

DD Data deficient

SN Sierra Nevada

## Taxonomic ranks

***Kingdom*:** Plantae

***Phylum*:** Magnoliophyta, Pteridophyta

***Class*:** Liliopsida (Monocotyledones), Magnoliopsida (Dicotyledones), Psilotopsida

***Order*:** Apiales, Asterales, Asparagales, Boraginales, Brassicales, Caryophyllales, Celastrales, Ericales, Fabales, Gentianales, Lamiales, Liliales, Malpighiales, Myrtales, Ophioglossales, Poales, Ranunculales, Rosales, Saxifragales

***Family*:** Apiaceae, Asparagaceae, Asteraceae, Boraginaceae, Brassicaceae, Campanulaceae, Caryophyllaceae, Celastraceae, Crassulaceae, Cyperaceae, Ericaceae, Fabaceae, Gentianaceae, Juncaceae, Lentibulariaceae, Liliaceae, Linaceae, Onagraceae, Ophioglossaceae, Plantaginaceae, Poaceae, Portulacaceae, Polygonaceae, Ranunculaceae, Rosaceae, Rubiaceae, Scrophulariaceae, Violaceae

***Genus*:** *Agrostis*, *Anthericum*, *Arenaria*, *Botrychium*, *Bromus*, *Campanula*, *Carex*, *Cerastium*, *Cirsium*, *Dactylis*, *Draba*, *Eleocharis*, *Epilobium*, *Erophila*, *Eryngium*, *Euphrasia*, *Festuca*, *Gagea*, *Galium*, *Gentiana*, *Gentianella*, *Herniaria*, *Juncus*, *Linaria*, *Lotus*, *Luzula*, *Meum*, *Montia*, *Myosotis*, *Nardus*, *Parnassia*, *Paronychia*, *Phleum*, *Pinguicula*, *Plantago*, *Poa*, *Potentilla*, *Radiola*, *Ranunculus*, *Rumex*, *Sagina*, *Scorzoneroides*, *Sedum*, *Silene*, *Spergularia*, *Stellaria*, *Thlaspi*, *Trifolium*, *Vaccinium*, *Veronica*, *Viola*

## Spatial coverage

### General spatial coverage

**Figure 4.**
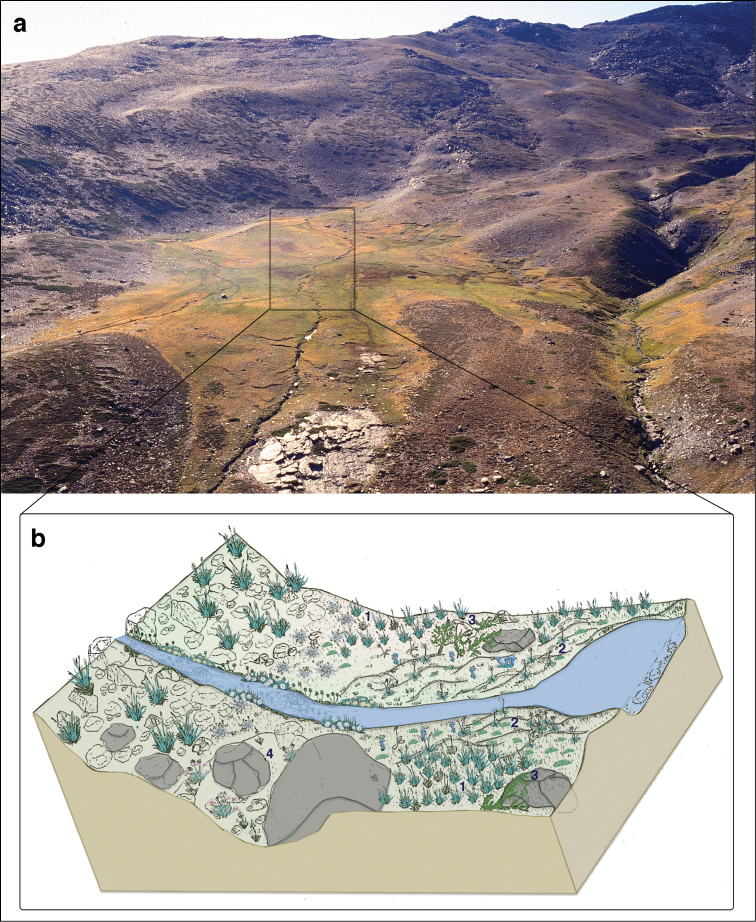
(**a**) Panoramic view of the borreguil of San Juan valley. The particular zonation of this ecosystem depending on soil moisture is reflected in the different colours of the borreguil. (**b**) Schematic representation of the vegetal communities forming the borreguiles, including dry borreguil (**4** *Armerio-Agrostietum nevadensis*), dense grassland (**1** *Nardo-Festucetum ibericae*), incipient peat formations (**2** *Ranunculo-Caricetum intrincatae*) and variants of borreguil in promontory areas (**3** *Ranunculo-Vaccinietum uliginosi*). Modified from Losa-Quintana et al. (1986). Picture: JM Martín-Martín.

The present dataset covers the Mediterranean high-mountain meadows ecosystems (known locally as *borreguiles*), which is considered a singular ecosystem of the Sierra Nevada (Bonet et al. 2010) (for a description of Sierra Nevada see study area of the Project section). Borreguiles are conditioned by the snow dynamics and are potentially sensitive to changes in water availability and temperature (Martínez-Parras et al. 1987, Fernández-Casas 1974). This ecosystem occupies an altitudinal range between 2200 and 3000 m a.s.l. and its distribution is determined by accumulation of the meltwater (Fernández-Casas 1974). Although it represents only 1.4% of this mountain range (1125 ha), it has a high rate of plant endemicity (Table 1) (Bonet et al. 2010, APMM 2013). The borreguiles are included in the Annex I of the Habitats Directive (EU habitat code 6230) (Bartolomé et al. 2005, Rigueiro et al. 2009). This ecosystem lies over hydromorphic soils that develop around mountain lakes, streams, depressions and glacial valleys. The overall appearance of borreguiles in summer is intense green, contrasting with the yellowish colour of the surrounding psychroxerophilic grasslands (Figure 4).

This ecosystem contains several plant communities arranged as parallel bands in relation to natural water courses (Molero-Mesa 1999, Lorite 2001, Lorite et al. 2003, Sánchez-Gutiérrez and Pino 2004) (Figure 4). The floristic composition of these communities depends on moisture content of the substrate. First, on some moist soil, as a transition from dry grasslands to the borreguiles themselves, there is a medium coverage grassland called *dry borreguil* (*Armerio-Agrostietum nevadensis*). It hosts species such *Agrostis nevadensis*, *Plantago nivalis*, *Ranunculus acetosellifolius*, *Thymus serpylloides* or Arenaria tetraquetra subsp. amabilis (among others) (Losa-Quintana et al. 1986, Lorite 2001). Then *dense grassland* appears, located in areas with constant moisture throughout the summer and deep soils. As typical species of this community (*Nardo-Festucetum ibericae*) include *Nardus stricta*, *Festuca iberica*, *Scorzoneroides microcephala*, Lotus corniculatus subsp. glacialis, *Luzula spicata*, *Ranunculus demissus*, and *Campanula herminii*. Moreover, in the promontory areas appears a variation of the borreguil (*Ranunculo-Vaccinietum uliginosi*) enriched with the presence of Vaccinium uliginosum subsp. nanum. In places under constant flooding and still waters until fall, the optimum conditions of oxygen deprivation exist for *incipient peat formations*. These communities (*Ranunculo-Caricetum intrincatae*) are characterized by the presence of species such as *Carex nigra*, *Eleocharis quinqueflora*, *Carex echinata*, *Carex nevadensis*, *Juncus articulatus*, *Ranunculus angustifolius*, *Pinguicula nevadensis* or *Festuca frigida*.

In addition to its high ecological value, this ecosystem plays an important role in transhumance livestock systems (Robles et al. 2009). These are pastures with a high nutritive value and with the greatest forage production of the Sierra Nevada ecosystems (Boza et al. 2007, González-Rebollar 2006, Robles et al. 2009, APMM 2013). This is important because they act as a trophic reserve for livestock in summer (Fernández-Casas 1974, Robles 2008). However, the abandonment of uses linked to this practice has tended to reduce the surface area of these ecosystems and consequent overloading of neighbouring areas (González-Rebollar 2006, Robles 2008).

### Coordinates

36°52'12"N and 37°21'36"N Latitude; 3°41'24"W and 2°33'36"W Longitude

### Temporal coverage

May 1988 – Oct 2013

### Parent collection identifier

NA

### Collection name

Dataset of phenology of Mediterranean high-mountain meadows flora (Sierra Nevada, Spain)

### Collection identifier

http://www.gbif.es:8080/ipt/resource.do?r=borreguiles

## Methods

### Study extent description

We selected one of the most representative borreguiles of Sierra Nevada (for more info about borreguiles ecosystems see “General spatial coverage” section), located at San Juan river basin (Guejar-Sierra; Granada, Spain) (Figure 1c). The catchment area is nearly 1325 ha. and the basin was formed by glacial erosion of the bedrock (mica schists) and presents a valley with U-shaped (Martín-Martín et al. 2010). This meadow, which originated about 2000 years ago (Esteban 1996), occupies an area of approximately 100 ha.

### Sampling description

**Figure 5.**
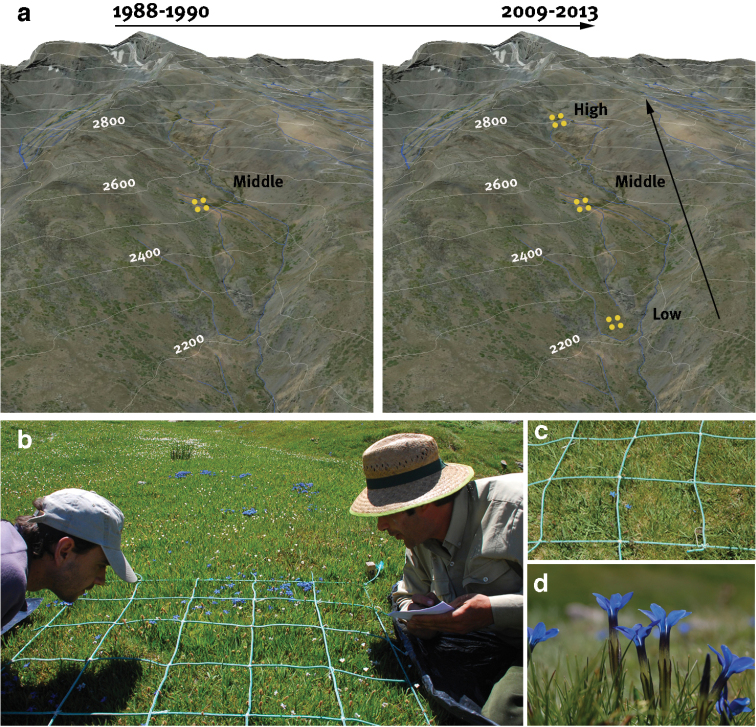
Schema of the sampling design. **a** Different sampling plots were distributed along an altitudinal gradient. For the middle-altitude locality the plots were sampled in two periods: 1988–1990 and 2009–2013. View of a sampling plot of 1 × 1 m (**b**) that was divided into quadrats of 25 × 25 cm to facilitate counting (**c**) and to record the cover-abundance and the number of individuals in flowering (**d**) or in fruit phenophase.

We sampled at three localities along an altitudinal gradient (Figure 5a): one at Prado de la Mojonera (Low Altitude; around 2200 m a.s.l.) and two at Hoya del Moro (middle and high altitude; 2430–2550 m a.s.l. and around 2775 m a.s.l., respectively). For each locality, the sampling was performed every 15 days during the free-snow period once a year from 1988–1990 and from 2009 to 2013. For the middle altitude locality, we have data from two periods: 1988–1990 and 2009–2013. For low- and high-altitude locations, we have data from 2009–2013 period.

At each locality, permanent plots of 1 × 1 m were distributed to cover the different types of borreguiles. In each plot, a floristic inventory was made. The presence/absence and an estimation of abundance-coverage using the Braun-Blanquet cover-abundance scale (Braun-Blanquet 1964) were recorded for each taxa (Figure 5b). We also counted the number of individuals belonging to the three main phenological phases (phenophase) established: vegetative phenophase, reproductive phenophase (flowering) and seed phenophase. The plots were divided into quadrats of 25 × 25 cm to facilitate counting (Figure 5c) (Sánchez-Rojas 2012).

### Method step description

All data were stored in a normalized database and incorporated into the Information System of Sierra Nevada Global-Change Observatory. Taxonomic and spatial validations were made on this database (see Quality-control description). A custom-made SQL view of the database was performed to gather occurrence data and other variables associated with some occurrence data, specifically:

- Flowering abundance: number of flowering individuals per square meter
- Fruit abundance: number of individuals in fruiting period per square meter
- Cover: the percentage of cover per taxon. The value represents a transformation of Braun-Blanquet cover-abundance scale (van der Maarel 1979, 2007)

The occurrence and measurement data were accommodated to fulfil the Darwin Core Standard (Wieczorek et al. 2009, 2012). We used Darwin Core Archive Validator tool (http://tools.gbif.org/dwca-validator/) to check whether the dataset meets Darwin Core specifications. The Integrated Publishing Toolkit (IPT v2.0.5) (Robertson et al. 2014) of the Spanish node of the Global Biodiversity Information Facility (GBIF) (http://www.gbif.es:8080/ipt) was used both to upload the Darwin Core Archive and to fill out the metadata.

The Darwin Core elements for the occurrence data included in the dataset are: occurrenceId, modified, language, basisOfRecord, institutionCode, collectionCode, datasetName, catalogNumber, scientificName, kingdom, phylum, class, order, family, genus, specificEpithet, infraspecificEpithet, scientificNameAuthorship, continent, country, countryCode, stateProvince, county, locality, minimumElevationInMeters, maximumElevationInMeters, decimalLongitude, decimalLatitude, coordinateUncertaintyinMeters, geodeticDatum, recordedBy, DayCollected, MonthCollected, YearCollected, EventDate.

For the measurement data, the Darwin Core elements included are: id, measurementID, measurementType, measurementValue, measurementAccuracy, measurementUnit, measurementDeterminedDate, measurementDeterminedBy, measurementMethod, measurementRemarks.

### Quality control description

The sampling plots were georeferenced using a Garmin eTrex Legend GPS (ED1950 Datum) with an accuracy of ±5 m. We also used colour digital orthophotographs provided by the Andalusian Cartography Institute and GIS (ArcGIS 9.2; ESRI, Redlands, California, USA) to verify that the geographical coordinates of each sampling plot were correct (Chapman and Wieczorek 2006).

The specimens were taxonomically identified using *Flora Iberica* (Castroviejo et al. 1986-2005, Castroviejo 2001) and others reference floras: *Flora de Andalucía Oriental* (Blanca et al. 2011), *Flora Vascular de Andalucía Oriental* (Valdés et al. 1987) and *Flora Europaea* (Tutin et al. 1964–1980). The scientific names were checked with databases of International Plant Names Index (IPNI 2013) and Catalogue of Life/Species 2000 (Roskov et al. 2013). We also used the R packages taxize (Chamberlian and Szocs 2013, Chamberlain et al. 2014) and Taxostand (Cayuela and Oksanen 2014) to verify the taxonomical classification.

We also performed validation procedures (Chapman 2005a, 2005b) (geopraphic coordinate format, coordinates within country/provincial boundaries, absence of ASCII anomalous characters in the dataset) with DARWIN_TEST (v3.2) software (Ortega-Maqueda and Pando 2008).

## Dataset description

### Object name

Darwin Core Archive Phenology of Mediterranean high-mountain meadows flora (Sierra Nevada, Spain).

**Character encoding:** UTF-8

**Format name:** Darwin Core Archive format

**Format version:** 1.0

**Distribution:** http://www.gbif.es:8080/ipt/resource.do?r=borreguiles

**Publication date of data:** 2014-12-03

**Language:** English

**Licenses of use:** This *Dataset of Phenology of Mediterranean high-mountain meadows flora (Sierra Nevada, Spain)* is made available under the Open Data Commons Attribution License: http://www.opendatacommons.org/licenses/by/1.0

**Metadata language:** English

**Date of metadata creation:** 2014-11-18

**Hierarchy level:** Dataset
